# High-dose stereotactic body radiotherapy correlates increased local control and overall survival in patients with inoperable hepatocellular carcinoma

**DOI:** 10.1186/1748-717X-8-250

**Published:** 2013-10-27

**Authors:** Won Il Jang, Mi-Sook Kim, Sun Hyun Bae, Chul Koo Cho, Hyung Jun Yoo, Young Seok Seo, Jin-Kyu Kang, So Young Kim, Dong Han Lee, Chul Ju Han, Jin Kim, Su Cheol Park, Sang Bum Kim, Eung-Ho Cho, Young Han Kim

**Affiliations:** 1 Department of Radiation Oncology, Korea Institute of Radiological & Medical Science, Seoul, Republic of Korea; 2 Department of Radiation Oncology, Kyung Hee University Gangdong Hospital, Seoul, Republic of Korea; 3 Division of Heavy Ion Clinical Research, Korea Institute of Radiological & Medical Science, Seoul, Republic of Korea; 4 Cyberknife Center, Korea Institute of Radiological & Medical Science, Seoul, Republic of Korea; 5 Department of Internal Medicine, Korea Institute of Radiological & Medical Science, Seoul, Republic of Korea; 6 Department of Surgery, Korea Institute of Radiological & Medical Science, Seoul, Republic of Korea; 7 Department of Radiology, Korea Institute of Radiological & Medical Science, Seoul, Republic of Korea

**Keywords:** Hepatocellular carcinoma, Radiotherapy, Stereotactic body radiotherapy, Dose–response relationship, Dose-survival relationship

## Abstract

**Background:**

Recent studies using stereotactic body radiotherapy (SBRT) for hepatocellular carcinoma (HCC) have reported high tumor response and local control. However, the optimal SBRT dose remains unknown, and it is still not clear whether a dose response relationship for local control (LC) and overall survival (OS) exist or not. We performed this study to determine whether a dose response relationship for LC and OS is observed in SBRT for inoperable HCC.

**Methods:**

Between 2003 and 2011, 108 patients with HCC were treated with SBRT. All patients were unsuitable for surgery or local ablation and had incomplete response to transarterial chemoembolization. Eighty-two patients with a longest tumor diameter (LD) less than or equal to 7.0 cm who were treated with 3-fraction SBRT and were analyzed. This cohort comprised 74 Child-Turcotte-Pugh (CTP) class A patients and 8 CTP class B7 patients. The median LD was 3.0 cm (range, 1.0–7.0 cm), and the median dose was 51 Gy (range, 33–60 Gy).

**Results:**

LC and OS rates at 2 years after SBRT were 87% and 63%, respectively, with a median follow-up duration of 30 months for all patients. The 2-year LC/OS rates for patients treated with doses of > 54, 45–54, and < 45 Gy were 100/71, 78/64, and 64%/30%, respectively (p = .009/p < .001). Multivariate analysis revealed that the SBRT dose (p = .005) and Barcelona Clinic Liver Cancer stage (p = .015) were significant prognostic factors for OS. Correlation analysis revealed a positive linear relationship between the SBRT dose and LC (p = .006, R = .899)/OS (p = .002, R = .940) at 2 years. Based on the tumor-control probability model, a dose of 54.8 Gy provides 2-year LC with a 90% probability. Five patients experienced grade 3 or higher gastrointestinal toxicity, and 6 had deteriorating of CTP score by greater than or equal to 2 within 3 months of SBRT.

**Conclusions:**

This study demonstrated a dose response relationship for LC and OS with SBRT for HCC. Higher LC rates resulting from an increased dose may translate into survival benefits for patients with HCC.

## Background

Liver cancer is the sixth most frequently diagnosed cancer worldwide, but the it was the second most frequent cause of cancer death in 2008 [1]. The treatment of choice for hepatocellular carcinoma (HCC) is surgery, but less than 20% of patients are suitable for surgery [2-5]. For patients with inoperable HCC, radiofrequency ablation (RFA) and other ablative therapies achieve excellent local control (LC) for small tumors. However, not all patients are suitable for these local therapies because of a large tumor size, tumor location, unmanageable coagulopathy, or invisibility on ultrasonography [6-8]. For patients with HCC unsuitable for local ablative therapies, transarterial chemoembolization (TACE) had been widely used as the first line non-curative therapy [9]. Radiotherapy (RT) has typically not been considered a frontline treatment for HCC due to the lower tolerance of the whole liver to RT [10]. However, some recent studies reported favorable outcomes for three-dimensional conformal radiotherapy (3D-CRT) for HCC [11-14].

Stereotactic body radiotherapy (SBRT) is an external beam RT method used to very precisely deliver a high dose of radiation to an extracranial target within the body using either a single fraction or a small number of fractions [15]. As the liver obeys the parallel architecture model of radiobiology, the risk of radiation-induced liver disease (RILD) is generally proportional to the mean dose of radiation delivered to normal liver tissue [16-18]. Several studies using SBRT for liver tumors have been performed, and these studies reported high tumor response and LC rates [19-24]. We previously reported our results from phase I and II trials of SBRT for HCC and observed high LC rates and low severe toxicity rates [25,26]. Due to the small number of patients in each study, we were unable to determine the optimal dose for LC, or clarify a dose response relationship for local control and overall survival. In this study, we expanded our previous study to include more cases of SBRT for HCC and analyzed additional data to determine whether a dose response relationship for local control and overall survival is observed in SBRT for inoperable HCC.

## Methods

### Patients

Between March 2003 and February 2011, 108 patients with 122 HCC lesions were treated with SBRT, and the medical records of these patients were retrospectively reviewed. This study was approved by the institutional review board of our institution. These patients included those who previously participated in phase I (n = 38) and II (n = 47) trials and additional patients (n = 23) who refused to participate in prospective clinical trials. The study eligibility criteria were previously reported [25,26]. All patients had diseases unsuitable for surgery or local ablation and underwent TACE before SBRT. Only patients with incomplete response to TACE were treated with SBRT and enrolled in this study. Incomplete response to TACE was defined as incomplete tumor filling by the lipiodol-doxorubicin mixture used by response evaluation computed tomography (CT) images at the 1 month after adequately performed TACE or increasing alpha-fetoprotein level. All patients provided written informed consent after receiving an explanation concerning the possible benefits and complications of SBRT versus 3D-CRT. The 23 patients who refused to participate in clinical trials also selected SBRT and they were treated using the same method as those in the clinical trials. In this study, only lesions treated with SBRT in 3 fractions were analyzed to avoid introducing confounding effect or errors from converting the biologically equivalent dose (BED) between different fractionations. In addition, only lesions with a longest tumor diameter (LD) ≤ 7.0 cm were analyzed to avoid introducing bias from the inclusion of extremely large tumors. Twenty-six patients with 27 lesions were excluded for following reasons: (1) SBRT with more than 3 fractions in 22 patients with 23 lesions and (2) LD > 7.0 cm in 4 patients with 4 lesions. In total, 82 patients with 95 lesions were analyzed. The patient and tumor characteristics are shown in Table 1. Fifty-four (66%) patients were diagnosed with recurrent disease. A recurrent disease is defined as a return of cancer after curative treatment, that is surgery, local ablations, and after a period of time during which the cancer cannot be detected. Seventy-four (90%) patients had Child-Turcotte-Pugh (CTP) class A disease, and 8 (10%) had CTP class B7 disease. All patients underwent TACE before SBRT and 44 patients (54%) underwent 1 or 2 sessions. Eleven patients (13%) had multiple hepatic lesions and all of the lesions were treated with SBRT in 1 session. The median LD was 3.0 cm (range, 1.0–7.0 cm). All patients received no further treatment after SBRT, if no progression was observed. The patients with intrahepatic or extrahepatic progression received customized salvage treatment. Thirty-one patients were treated with TACE, 10 with RFA, 1 with percutaneous ethanol injection, 2 with surgical resection, 1with liver transplantation, 12 with sorafenib, 2 with chemotherapy othan than sorafenib, and 6 with SBRT at progression.

**Table 1:** Patient and tumor characteristics

**Characteristics**	**No. of patients (%)**	
Age (years)	Median (Range)	60 (39–79)
	≤ 60	43 (52)
	> 60	39 (48)
Gender	Male	60 (73)
	Female	22 (27)
Etiology	Hepatitis B virus	55 (67)
	Hepatitis C virus	7 ( 9)
	Others	20 (24)
Diagnosis history at SBRT	Initially diagnosed	28 (34)
	Diagnose as recurrence	54 (66)
No. of previous TACE sessions	≤ 2	44 (54)
	> 2	38 (46)
Alpha-fetoprotein (IU/ml)	Median (Range)	14.0 (1.3–6055)
	≤ 200	60 (73)
	> 200	22 (27)
Child-Turcotte-Pugh score	A5	61 (74)
	A6	13 (16)
	B7	8 (10)
Portal vein tumor thrombosis	Yes	8 (10)
	No	74 (90)
No. of tumor	1	71 (87)
	2*	9 (11)
	3*	2 (2)
AJCC stage	T1	44 (54)
	T2	16 (19)
	T3	22 (27)
BCLC stage	A	43 (53)
	B	24 (29)
	C	15 (18)
Okuda stage	I	64 (78)
	II	18 (22)
CLIP score	0	39 (48)
	1	32 (39)
	2	11 (13)
Longest diameter (cm)	Median (Range)	3.0 (1.0–7.0)
	1.0-2.0	10 (12)
	2.1-3.0	23 (28)
	3.1-4.0	22 (27)
	4.1-5.0	13 (16)
	5.1-6.0	4 (5)
	6.1-7.0	10 (12)
SBRT dose (Gy)	Median (Range)	51 (33–60)
	< 45	32 (39)
	45–54	40 (49)
	> 54	10 (12)

*Abbreviations*: *SBRT* stereotactic body radiotherapy, *TACE* transarterial chemoembolization, *AJCC* American Joint Committee on Cancer, *BCLC* Barcelona Clinic Liver Cancer, *CLIP* Cancer of the Liver Italian Program.

*All multiple lesions were treated with *SBRT* in one session.

### Stereotactic body radiotherapy

The SBRT technique used at our institution has been previously described [25,26]. Briefly, patients were immobilized with a customized external vacuum-type (Vac-Loc; MedTec, Inc., Orange City, IA). Breathing-related tumor motion was minimized by abdominal compression using 4 belts [27]. Gold fiducials (4 mm x 0.8 mm) or lipiodol deposits in the tumor were used to mark tumors for SBRT. Daily image guidance using orthogonal ×-ray imaging or on-board CT was used to relocalize the target lesion before treatment delivery. A CT image was taken with a slice thickness of 2 mm at 3 seconds per slice. These relatively slow CT images included the respiratory movement of the target; therefore, the tumor volume used for planning was larger than the gross tumor volume (GTV) and was referred to as the internal target volume (ITV) [28,29]. The planning target volume (PTV) was defined as the ITV + 4 mm in the craniocaudal direction and the ITV + 2 mm in all other directions [30]. SBRT doses were prescribed at an isodose line (70–80% of the maximum dose) that covered at least 97% of the PTV.

The SBRT doses were escalated from 33 Gy in 3 fractions to 60 Gy in 3 fractions in our previous study. The final prescribed doses were 60 Gy in 3 fractions, but the dosages were reduced by 0.5 or 1 Gy per fraction until normal tissue constraints were allowed. We adopted the normal tissue constraint that at least 700 ml of normal liver (entire liver minus the cumulative GTV) should not receive a total dose ≥ 15 (phase I) or ≥ 17 Gy (phase II). For the spinal cord, the maximum dose should not exceed 18 (phase I) or 22 Gy and 18 Gy to 0.25 ml or less of irradiated volume (phase II). For the esophagus the maximum dose should not exceed 24 Gy. In addition, although other normal tissue constraints were not considered, dosages to the kidneys, intestine, and stomach were restricted to the lowest level possible. SBRT was administered in a 3-fraction course over no more than 14 elapsed days.

### Evaluation and statistical analysis

Treatment response was assessed using modified Response Evaluation Criteria in Solid Tumors [31]. Toxicities were graded according to the National Cancer Institute Common Terminology Criteria for Adverse Events (NCI-CTCAE) version 4.0. Classic RILD was defined as the presence of anicteric ascites and an at least twofold elevation in alkaline phosphatase relative levels to the pretreatment value in the absence of tumor progression [32]. Non-classic RILD was defined as elevated transaminase levels (> 5× the upper limit of normal) or NCI-CTCAE grade 4 levels in patients with baseline levels more than 5 times the upper limit of normal range, within 3 months after the completion of SBRT, or deterioration of the CTP score by ≥ 2, in the absence of classic RILD [33]. LC was defined as no tumor progression during follow-up and determined via post-SBRT radiographic studies. LC and overall survival (OS) rates (from the first date of SBRT treatment) were calculated using the Kaplan-Meier method and intergroup comparisons were performed using the log-rank test. All factors with p-value lower than or equal to 0.1 in univariate analysis were subjected to multivariate analysis using a Cox proportional hazards regression model with a forward, stepwise procedure to determine whether factors acted independently. All statistical analyses were performed using SPSS statistical software (version 12.0; SPSS, Inc., Chicago, IL, USA), and p-values < 0.05 were considered statistically significant.

### Tumor control probability model

The tumor control end point was LC at the 2-year time point. Doses to individual lesions were divided into 6 bins; the x-axis represented the mean dose given in that bin, whereas the y-axis value represented the probability of LC. The tumor control probability (TCP) for each bin was calculated using the Kaplan-Meier method. Quantification of the dose response to the tumor was estimated using the logistic model as follows [34].

$$\mathrm{TCP}=\frac{1}{1+{\left(\frac{D_{50}}{D}\right)}^{4/\gamma }}$$

where D refers to the total dose, γ describes the slope of the curve, and D50 is the dose that achieves a TCP of 50% for the prescribed dose. D50 and γ were estimated by logit functions. The 95% confidence intervals (CIs) were found using the probability density function of normal distribution. The formula was implemented using MATLAB R2011b (The MathWorks Inc., Natick, MA).

## Results

### Local control

**Figure 1:**
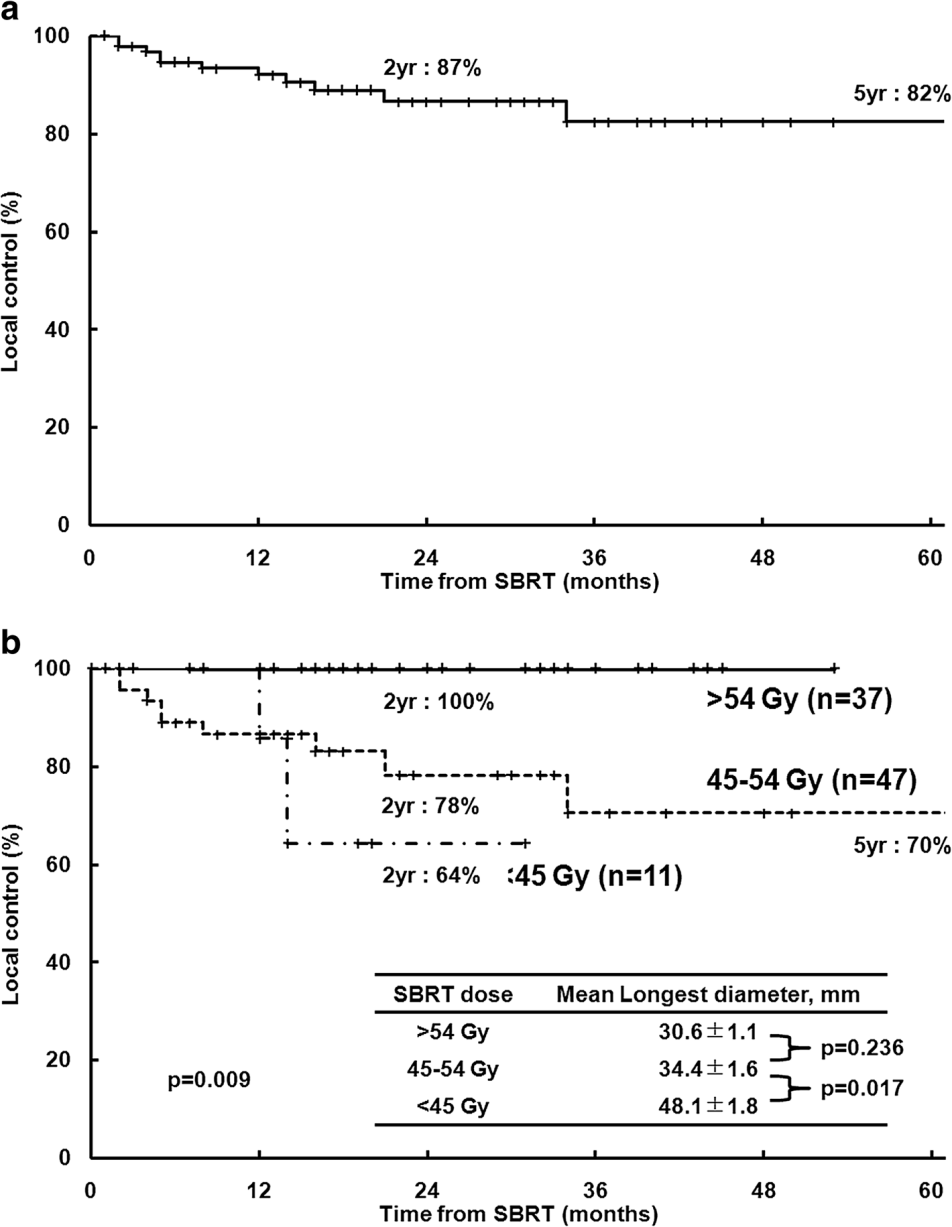
**Local control from the time of the first stereotactic body radiotherapy (SBRT) treatment. (a)** All lesions (n = 95); **(b)** By SBRT dose. yr, year.

**Figure 2:**
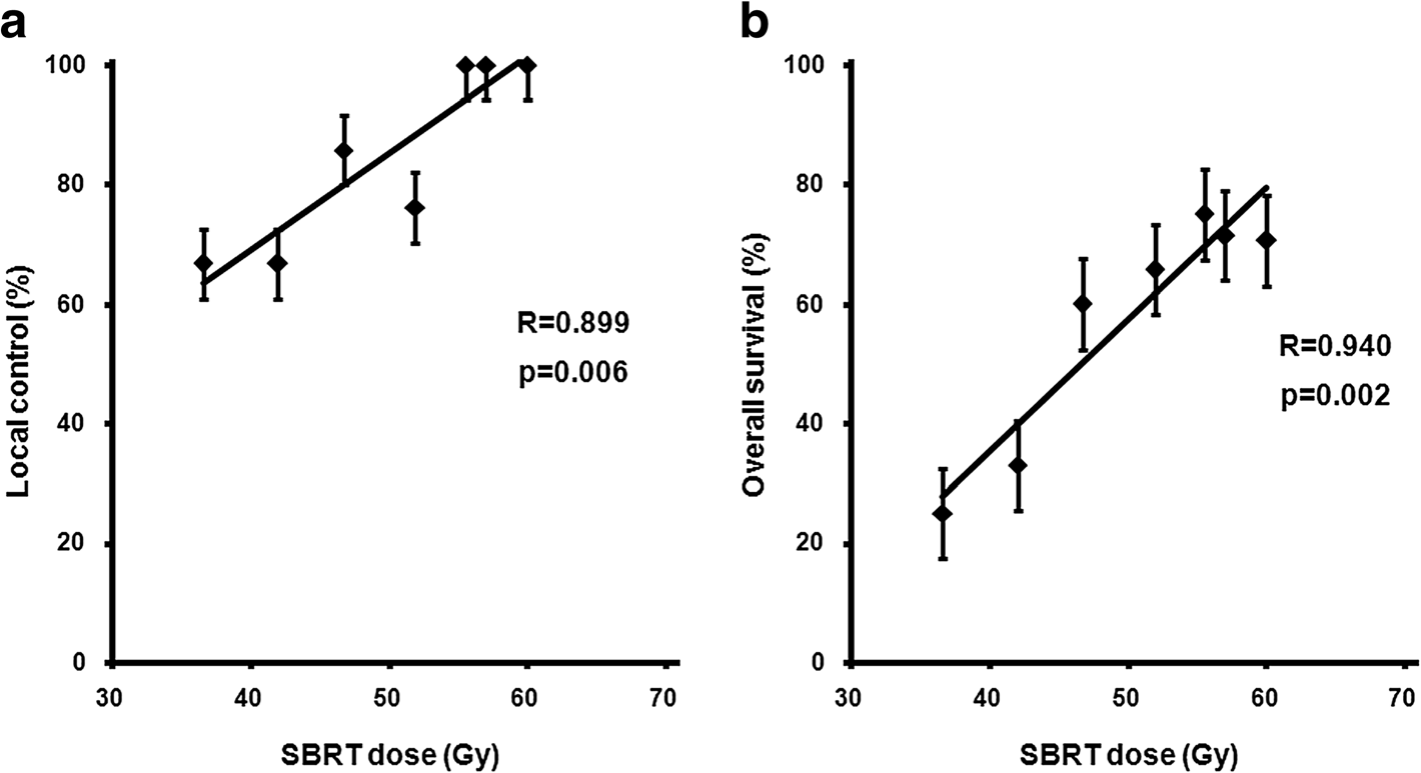
Correlation of the stereotactic body radiotherapy (SBRT) dose with (a) local control/ (b) overall survival.

The median follow-up duration after SBRT for all patients was 30 months (range, 4–81 months). In all lesions, the 2- and 5-year LC rates were 87 and 82%, respectively (Figure 1(a)). Univariate analysis identified age, number of previous TACE sessions, American Joint Committee on Cancer clinical T stage, Barcelona Clinic Liver Cancer (BCLC) stage, SBRT dose, and LD as significant prognostic factors for LC (Table 2). The 2-year LC rates for lesions treated with doses of > 54, 45–54, and < 45 Gy were 100, 78, and 64%, respectively (p = 0.009) Figure 1(b)). Correlation analysis revealed a positive linear relationship between the SBRT dose and LC at 2 years (p = 0.006, R = 0.899) (Figure 2(a)). Multivariate analysis confirmed that SBRT dose (as a continuous variable) (p = 0.027) and age (p = 0.026) were significant prognostic factors for LC.

**Table 2:** **Prognostic factors**: **univariate analysis**

**Characteristics**		** Local control**		**Overall survival**	
		**2**-**year (%)**	*P-**value**	**2**-**year (%)**	*P-**value**
Age (years)	≤ 60	79.5		59.6	
	> 60	92.8	0.019	66.3	NS
Gender	Male	83.3		57.8	
	Female	95.7	NS	76.8	NS
Diagnosis history at SBRT	Initially diagnosed	96.2		67.9	
	Recurrence	82.3	NS	60.3	NS
Child-Turcotte-Pugh score	A5, 6	85.3		60.1	
	B7	100.0	NS	87.5	NS
Portal vein tumor thrombosis	Yes	88.9		62.9	
	No	86.4	NS	62.5	NS
Alpha-fetoprotein (IU/ml)	≤ 200	88.4		66.3	
	> 200	81.5	NS	53.8	NS
AJCC stage	T1	93.7		71.3	
	T2, 3	73.2	0.031	53.8	NS
BCLC stage	A	93.7		74.7	
	B, C	78.8	0.018	51.0	0.011
Okuda stage	I	89.1		65.4	
	II	77.9	NS	55.0	NS
CLIP score	0	85.1		66.6	
	1, 2	88.6	NS	59.4	NS
Longest diameter (cm)	≤ 5.0	90.2		69.4	
	> 5.0	63.3	0.015	33.3	0.012
No. of previous TACE sessions	≤ 2	95.7		69.6	
	> 2	77.2	0.022	54.9	0.023
SBRT dose (Gy)	< 45	64.3		30.0	
	45–54	78.3	0.009	64.3	<0.001
	> 54	100.0		71.3	

*Abbreviations*: *SBRT* stereotactic body radiotherapy, *TACE* transarterial chemoembolization, *AJCC* American Joint Committee on Cancer, *BCLC* Barcelona Clinic Liver Cancer, *CLIP* Cancer of the Liver Italian Program; *NS* not significant (p > 0.05).

*P-value was calculated by log-rank test.

### Overall survival

**Figure 3:**
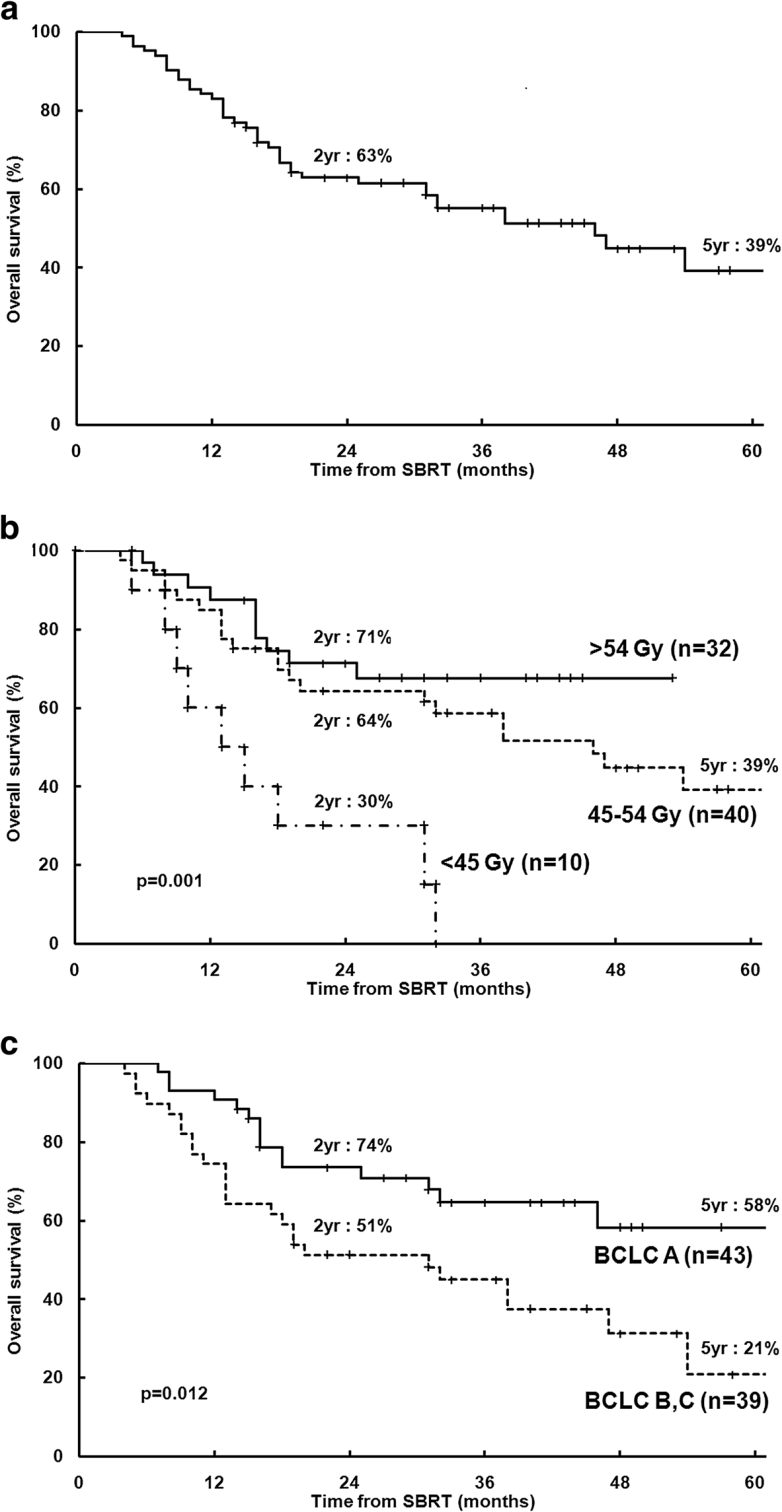
**Overall survival from the time of the first stereotactic body radiotherapy (SBRT) treatment. (a)** All patients (n = 82); **(b)** By SBRT dose; **(c)** By Barcelona Clinic Liver Cancer (BCLC) stage. yr, year.

In all patients, the 2- and 5-year OS rates were 63 and 39%, respectively (Figure 3(a)). Univariate analysis identified the number of previous TACE sessions, BCLC stage, SBRT dose, and LD as significant prognostic factors for OS (Table 2). The 2-year OS rates for patients treated with SBRT doses of > 54, 45–54, and < 45 Gy were 71, 64, and 30%, respectively (p = 0.001) (Figure 3(b)). The 2-year OS rates for BCLC A and B-C were 74 and 51%, respectively (p = 0.012) (Figure 3(c)). Correlation analysis revealed a positive linear relationship between the SBRT dose and OS at 2 years (p = 0.002, R = 0.940) (Figure 2(b)). Multivariate analysis confirmed that the SBRT dose (as a continuous variable) (p = 0.005) and BCLC stage (p = 0.015) were significant prognostic factors for OS. To determine the effect of a local failure on survival, local failure was analyzed as a prognostic factor. Univariate analysis revealed that the 2-year OS rates for patients with and without local failure were 27% and 68%, respectively (p < 0.001). In 32 patients with 37 lesions with dose > 54 Gy, LC and OS rates at the time of the last follow-up (4.5 years) were 100% and 68%, respectively (Table 3).

**Table 3:** **Published studies of RFA and high**-**dose SBRT group of the present study**

**Study**, **year**	**Treatment**	**Study type**	**Number**	**Tumor size (cm)**	**Local control (%)**	**Overall survival (%)**	**Severe toxicity (%)**
Shiina 2005 (7)	RFA	RCT	118	Median 2.2	98	74 (4-year)	5.1
Bouza 2009 (8)	RFA	Meta-analysis	396	Mean 2.6	93	62 (4-year)	4.1
Waki 2010 (38)	RFA	Retrospective	88	Median 1.8	95	70 (5-year)	5.7
Feng 2012 (39)	RFA	RCT	84	Mean 2.4	96	67 (3-year)	9.5
Shiina 2012 (6)	RFA	Retrospective	1170	Median 2.0 Mean 2.5	97	60 (5-year)	2.2
Present study(> 54 Gy)	SBRT	Retrospective	32 (Rec 53%)	Median 3.0 Mean 3.1	100	68 (4.5-year)	Bowel 3.1; Others 3.1

*Abbreviations*: *SBRT* stereotactic body radiotherapy, *RFA* radiofrequency ablation, *RCT* randomized controlled trial, Rec, recurrence.

Comparison between recent data concerning published *RFA* for hepatocellular carcinoma and high-dose (> 54 Gy) *SBRT* group of the present study. In these *RFA* series, all patients had initial disease and received *RFA* as the first treatment.

### Tumor control probability

**Figure 4:**
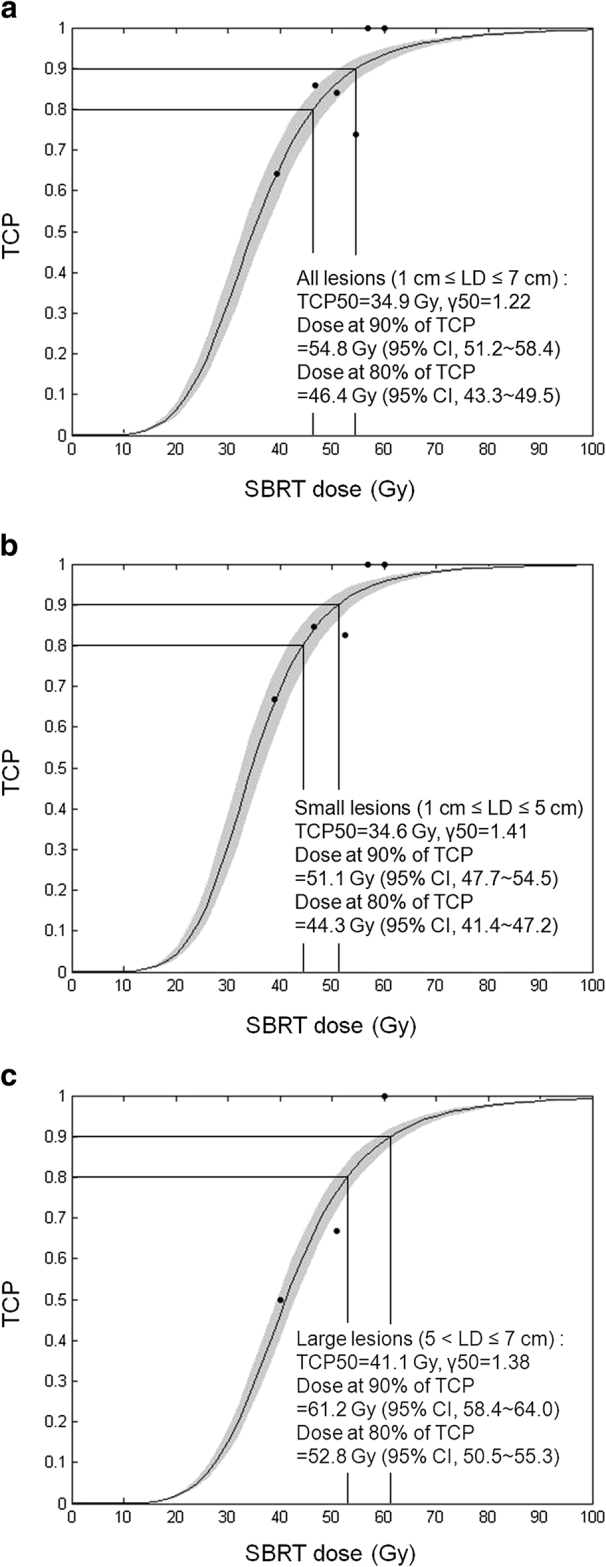
**Tumor control probability (TCP) curve by the 3**-**fraction stereotactic body radiotherapy (SBRT) dose. (a)** All lesions (n = 95); **(b)** 1.0 cm ≤ Longest diameter (LD) ≤ 5.0 cm (n = 83); **(c)** 5.0 cm < LD ≤ 7.0 cm (n = 12). CI, confidence interval.

Figure 4(a) shows the fitted TCP of the SBRT dose versus the 2-year LC for all 95 lesions as plotted by the following fitted parameters: γ50 = 1.22 and TCD50 = 34.9 (95% CI, 32.6-37.2). According to the TCP curve, doses of 54.8 Gy (95% CI, 51.2-58.4) and 46.4 Gy (95% CI, 43.3-49.5) provide 2-year LC with probabilities of 90% and 80%, respectively. Figures 4(b) and 4(c) show the fitted TCP of the SBRT dose versus the 2-year LC for 83 lesions with LD ≤ 5.0 cm and 12 lesions with LD > 5.0 cm, respectively.

### Toxicity

One patient (1%) experienced grade 3 hyperbilirubinemia, but this patient had pre-existing grade 1 hyperbilirubinemia. Two patients (2%) experienced grade 3 ascites that required paracentesis without evidence of disease progression and with normal alkaline phosphatase levels. No classic RILD was observed, but 6 patients (7%) experienced non-classic RILD (worsening of CTP score by ≥ 2 in all) within 3 months after SBRT. Excluding the patient with disease progression, 4 patients (5%) experienced non-classic RILD, and 3 of these patients were reversibly converted during long-term follow-up. One patient (1%) experienced grade 3 soft tissue toxicity in the right upper quadrant of the abdomen, and this patient had a large tumor (LD, 6.2 cm) near the skin. Five patients (6%) experienced grade 3 or 4 gastrointestinal (GI) toxicity as follows: gastroduodenal ulcer in 2 patients, colonic ulcer in 1 patient, and gastroduodenal perforation in 2 patients. Two patients with gastroduodenal perforation recovered after supportive care in one and primary repair in the other.

## Discussion

**Figure 5:**
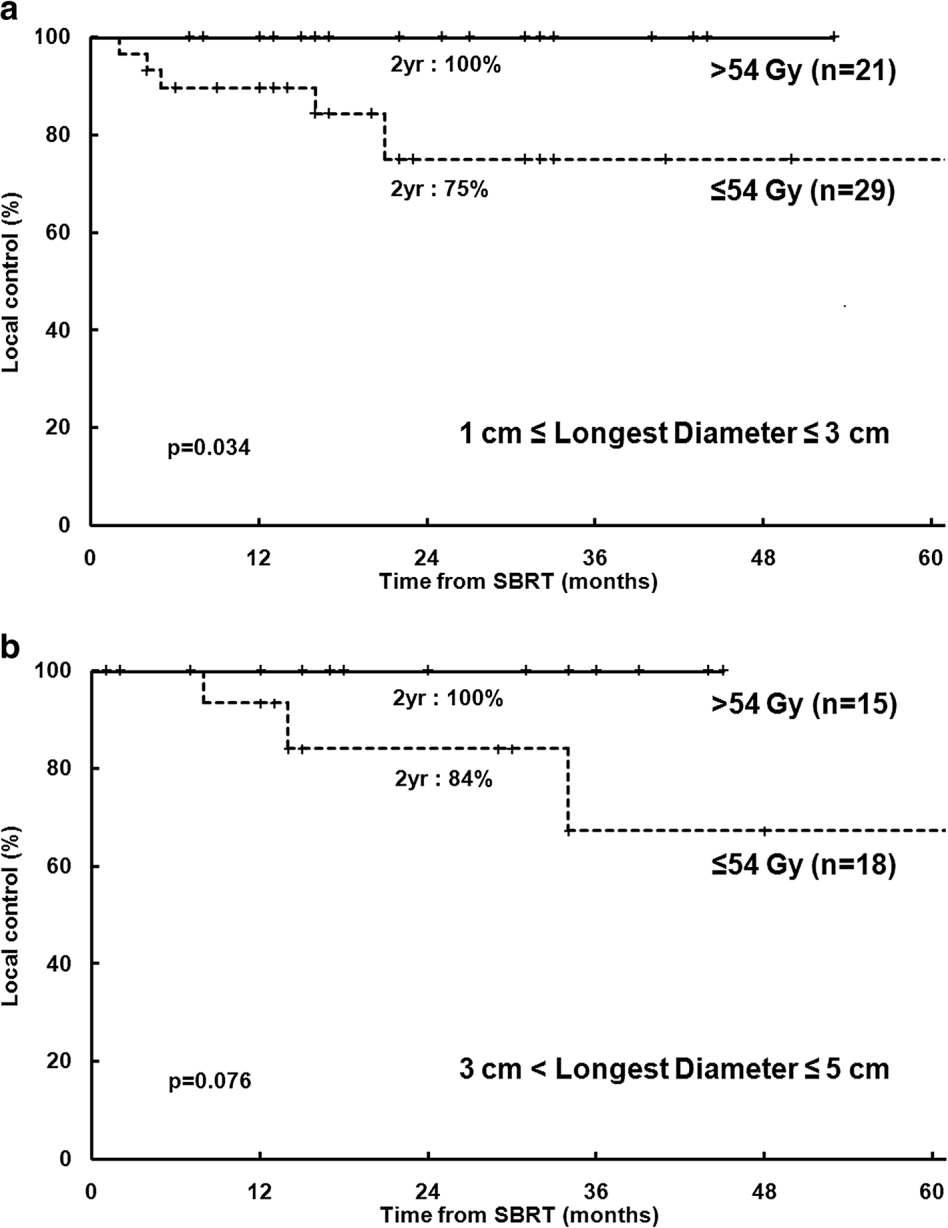
**Local control from the time of the first stereotactic body radiotherapy (SBRT) treatment. (a)** 1.0 cm ≤ Longest diameter (LD) ≤ 3.0 cm (n = 50); **(b)** 3.0 cm < LD ≤ 5.0 cm (n = 33). yr, year.

This study revealed a dose-control relationship for SBRT in 3 fractions for HCC. Higher SBRT doses resulted in higher LC rates. In particular, we analyzed only lesions treated with 3 fractions, and calculating the BED was not necessary; therefore, no confounding effect or error was introduced from converting the dose between different fractionations. More specifically, a SBRT dose > 54 Gy achieved LC rates of 100%. This result using > 54 Gy in 3 fractions is consistent with those found in the phase II study of SBRT for early stage non-small cell lung cancer (3-year LC of 98% with 54 Gy in 3 fractions) [35]. In this study, LD was a prognostic factor for LC in univariate analysis. Although statistical significance for LC was not observed in multivariate analysis, tumor size is generally an important prognostic factor in HCC [3-6]. The SBRT dose and tumor size are closely related due to normal liver dose-constraints. Therefore we performed stratified analysis according to LD to avoid the effect of tumor size, demonstrating that the SBRT dose remained significantly prognostic factor for LC (Figures 5(a), (b)). In the lesions with LD > 5 cm, only 1 patient had a SBRT dose > 54 Gy, and we could not carried out adequate statistical analysis for the difference in LC between the SBRT dose groups (≤ 54 Gy vs. > 54 Gy).

For optimal dose quantification, we adopted the logistic model. On the basis of the best-fitted TCP curve, doses of 54.8 Gy and 46.4 Gy produce 2-year LC with probabilities of 90% and 80%, respectively. These findings suggest that 54 Gy in 3 fractions is an acceptable dose to achieve LC for HCC lesions with LD ≤ 7.0 cm. However, the SBRT dose required to achieve the same LC rates may differ according to tumor size. We estimated the TCP curve after stratification by LD (≤ 5.0 cm vs. > 5.0 cm). For lesions with LD ≤ 5.0 cm or > 5.0 cm, the estimated SBRT doses to provide 2-year LC with a probability of 90% were 51.1 (95% CI, 47.7–54.5) and 61.2 Gy (95% CI, 58.4–64.0), respectively.

To date, no prospective randomized study has compared dose-fractionation regimens in SBRT for HCC. We suggested a SBRT dose of 54 Gy in 3 fractions by fitted TCP curve and survival analysis. In an Indiana University study, the prescribed doses were 48 Gy in 3 fractions for patients with CTP class A and 40 Gy in 5 fractions for those with CTP class B. In that study, the 2-year LC rate was 90% [21]. In a Belgium study, the prescribed dose was 45 Gy in 3 fractions, and the 2-year LC rate was 95% [22]. The results of these studies are comparable with those of the lesions that received higher doses in our study, despite use of slightly lower doses in the above mentioned studies. These observations may be attributable to the additional margin to the GTV. In the Indiana University study, CTV expansion was not performed, and the PTV was defined as GTV + 5–10 mm. In the Belgium study, the CTV was defined as the GTV + 10 mm in all directions the within liver and the PTV as CTV + 1.5 mm margin. In our institution, CTV expansion was not performed and the PTV was defined as GTV + 2–4 mm. In SBRT, the differences in the additional margin to the GTV affect dose-distribution. Even without a GTV expansion, the surrounding 0.5 cm may be adequately covered by the SBRT prescription. In fact, the total dose to cover the GTV may be similar among 3 institutions. When we reviewed our plan, PTVs with additional margins of 5–10 mm or 11 mm were adequately covered by isodose lines of 48 Gy and 45 Gy, respectively. Therefore, to compare the studies of SBRT for HCC, an additional margin and a prescribed isodose line should be considered. Comparisons using a dose covering the GTV or maximum dose may be suggested as appropriate methods. Furthermore, we think that consensus or standardization of the SBRT technique for HCC, such as defining of additional margin to GTV and prescribed isodose line, is needed.

Although it appears that the tumor response increases with increasing radiation doses, it is difficult to demonstrate a dose-relationship for OS because many complicated factors are involved in determining survival. This is especially true in HCC because most of patients have liver cirrhosis, and minimizing the deterioration of liver function induced by treatment is critical. Further, as many patients who experienced intrahepatic tumor recurrence after treatment exhibited a complete response, preserving liver function for further treatment is important for prolonging OS. In patients with HCC treated by 3D-CRT, RILD is a relatively common severe toxicity [11-14,36]. However, classic RILD was not reported after SBRT and we also observed no classic RILD, perhaps due to the substantial difference in the normal liver dose distribution between 3D-CRT and SBRT [20-26]. On the other hand, we observed non-classic RILD in 6 patients (7%) within 3 months after SBRT. Excluding the patient with disease progression, 4 patients (5%) experienced non-classic RILD. Additionally, if late toxicity can influence survival, the fact that 75% of patients who experienced non-classic RILD without disease progression were reversibly converted during long-term follow-up duration is encouraging. Due to the high LC rate and little liver deterioration induced by treatment, in our study, the SBRT dose as a continuous variable was a prognostic factor for OS in multivariate analysis.

Overall 5 patients (6%) experienced grade 3 or 4 gastrointestinal toxicity. These patients had the lesion in close proximity (within 0–0.4 cm) to the GI tract and relatively high doses were delivered to the GI tract. The maximum doses to GI tract of these patients were 42, 45, 55, 55, and 60 Gy, respectively. Three patients had pre-SBRT gastroduodenal ulcer confirmed by esophagogastroduodenoscopy. We previously performed studies about severe gastroduodenal and intestinal toxicity after SBRT using 3 fractions for abdominopelvic malignancies and reported their results [37,38]. In the study about gastroduodenal toxicity, we suggested that Dmax is a valuable predictor of severe gastroduodenal tocivity. A history of ulcer before SBRT should be carefully considered as a clinical predictor, especially in patients who receive a high dose to gastroduodenum [37]. In the study about intestinal toxicity, we V25 is a valuable predictor of severe intestinal toxicity. And SBRT would be conducted with a treatment interval of at least 48 hours if possible [38].

LC and OS rates in SBRT for HCC patients with dose > 54 Gy were excellent and potentially equivalent to those treated by RFA [6-8,39,40] (Table 3). Of course, because of differences in baseline prognostic features between the patients, direct comparison between SBRT and RFA has been limited. In most RFA series, the patients had initial disease and received RFA as the first treatment. However, in this study, 53% of the patients had recurrent disease and all patients had an incomplete response to prior TACE. Irrespective of these potential unfavorable prognostic features, the outcomes were comparable with those of RFA. Toxicities were also tolerable in SBRT for HCC. On the basis of these observations, a phase II trial of high-dose SBRT for the patients with small-sized HCC (≤ 5.0 cm) is ongoing in Korea. Furthermore, we expect randomized trials comparing SBRT and RFA for HCC to follow.

This study had some limitations. First, this study was not a randomized trial, and therefore, patients were not controlled with respect to variable prognostic factors. Nevertheless, all patients met the inclusion criteria of prospective studies and received SBRT with consistent technique. Therefore, selection bias can be controlled to a considerable degree. Second, the TCP curve was estimated using the logistic model. This result may be regarded as hypothesis generating and should be validated with clinical data. Third, only a small number of large tumors were included, and it may be inaccurate to apply our results to large tumors. We believe that our results are more powerful and reliable for small tumors rather than large tumors. In separate prospective SBRT trial for large HCC, we expect implemental results for large-sized HCC.

In conclusion, this study demonstrated a dose response relationship for LC and OS with SBRT for HCC. Higher LC rates resulting from higher doses may improve survival benefit for HCC. High-dose SBRT may be as effective and safe a treatment modality as RFA, a major nonsurgical ablative modality. Based on the TCP model, a dose of 54.8 Gy produces 2-year LC with a probability of 90%. To validate our results and obtain definitive conclusions, prospective studies in larger populations will be needed in the future.

## Competing interests

The authors declare that they have no competing interests.

## Authors’ contributions

WIJ contributed to study concepts, study design, literature research, definition of intellectual content, data acquisition, data analysis, data interpretation, statistical analysis, manuscript preparation and manuscript editing. MSK contributed to study concepts, study design, literature research, definition of intellectual content, data analysis, data interpretation, manuscript editing, and manuscript review. DHL performed the treatment planning and conducted all planning evaluations. SYK contributed to the estimation of tumor control probability model. All authors made substantial intellectual contributions to drafting the article, revising the article, data analysis, and data interpretation. All authors have read and approved the final manuscript.
